# The Early Stage of the COVID-19 Outbreak in Tunisia, France, and Germany: A Systematic Mapping Review of the Different National Strategies

**DOI:** 10.3390/ijerph18168622

**Published:** 2021-08-15

**Authors:** Khouloud Laffet, Fatma Haboubi, Noomene Elkadri, Rita Georges Nohra, Monique Rothan-Tondeur

**Affiliations:** 1 Nursing Sciences Research Chair, Laboratory Educations and Health Practices (LEPS), Université Sorbonne Paris Nord, (EA 3412), UFR SMBH, 93017 Bobigny, France; ritag.nohra@gmail.com (R.G.N.); rothan-tondeur@univ-paris13.fr (M.R.-T.); 2 Medical Research Directorate, The Ministry of Health of Tunisia, 1006 Bab Saadoun, Tunis 1006, Tunisia; fatma.habboubi@rns.tn (F.H.); noomene.elkadri@rns.tn (N.E.); 3 Hôtel-Dieu de France Hospital, Alfred Naccache Boulevard, Beirut 166830, Lebanon; 4 Faculty of Public Health, Branch II, Lebanese University, Fanar 248199, Lebanon; 5 Nursing Sciences Research Chair, Assistance Publique-Hôpitaux de Paris, 75005 Paris, France

**Keywords:** COVID-19, pandemic, health policy

## Abstract

The multitude of national strategies used against the COVID-19 pandemic makes it necessary to review and synthesize them in order to identify potential gaps and shortcomings, and to help prioritize future control efforts. This systematic mapping review is aimed at identifying the coronavirus pandemic management strategies adopted by France, Tunisia, and Germany during the early stage of the COVID-19 outbreak. A set of government websites in addition to the PubMed and Google Scholar databases were searched to identify scientific articles and institutional documents related to the national strategies of the three countries up until July 2020. The references included were mapped and narratively synthesized based on the pillars of the *Monitoring and Evaluation Framework of the Strategic Preparedness and Response Plan* defined by the World Health Organization. Of the 2765 records screened, 65 documents were included in the study. The analysis of these documents showed that Germany was the first country to implement mass screening of cases and that France was the first country to implement measures to impose general containment at the national level. It also showed that Tunisia was the only country to have imposed the confinement of passengers on repatriation flights in dedicated containment centers and at the expense of the state.

## 1. Introduction

Since December 2019, humanity has been facing a health crisis of unprecedented magnitude. Coronavirus disease 2019, or COVID-19, started as an outbreak in China and then spread rapidly around the world. On 11 March 2020, the World Health Organization announced that the situation reached the pandemic stage [1,2,3].

This pandemic is distinguished by three characteristics [4]:

- Its contagion and its high rate of spread caused the saturation of all health systems, even the most resilient. By 2 November 2020 the world had seen 46,049,978 cases and 1,201,442 deaths, including 516,774 new cases of contamination and 6088 new deaths in the previous 24 h [5].
- Its severity (20% of infected people develop a serious or critical form of the disease [4]).
- Its profound societal and economic consequences (over USD 220 billion has been lost in developing countries [6]).

Faced with this situation and the peculiarities of the rapid spread of COVID-19, each country applied a differentiated policy, which was always influenced by its degree of development, socio-economic situation, and political system [7,8,9].

The multitude and differences of strategies make it necessary to review and synthesize them in order to identify the most effective measures at the early stage of the pandemic and help prioritize future control efforts.

To this end, we carried out a systematic mapping review of three different national strategies, which are considered to be representative sample of what has been adopted in many countries.

Our choices were France, Germany, and Tunisia, for the representativeness of their strategies due to the countries’ cultural, political, and economic differences, and the availability and ease of access to data.

### 1.1. Background

#### 1.1.1. Socio-Economic Situations of Tunisia, Germany, and France

The socio-economic and political situation of countries has played a key role in implementing their national control strategies [10].

Countries’ degrees of preparedness have differed considerably, depending on their degrees of development. The provision of the resources and infrastructure needed to cope with the pandemic was one of the greatest challenges for all countries, and especially for developing countries whose health systems are far less efficient [10].

In our study we are interested in an African developing country, which is Tunisia, and two developed European countries but with different demographic and political characteristics, which are France and Germany.

Tunisia has a GDP of USD 38.79 billion, of which 7% is dedicated to health (2019). It experienced a popular revolution in 2011 that led to the establishment of a democracy. However, its economy, which was once considered one of the best performing in the developing world, is currently undergoing an unprecedented crisis. The protracted recession risks greatly reducing the ability of the authorities to respond to the various crises. Tunisia has 2.3 hospital beds, 1.3 doctors and 2.5 nurses per 1000 inhabitants [11].

The other two countries in the study are France and Germany, which are the two largest economic powers in the European Union with a GDP of USD 2716 billion for France and USD 3846 billion for Germany [11]. Each one of the two countries allocate about 11% of their gross domestic product to health care, which is the highest rate in Europe. France has 6.5 hospital beds and 11.5 nurses per 1000 inhabitants and Germany has 8.3 hospital beds and 13.2 nurses [12].

However, our choice of these two countries is due to the great differences between them in terms of political systems (Germany is a federal republic, while France is a republic with a semi-presidential system) and the organization of their health systems [13]. This allows us to have an overview of the role that a political system can play during a pandemic and health crisis.

Tunisia, France, and Germany also have different demographic properties (Table 1) [11].

#### 1.1.2. The COVID-19 Pandemic in Tunisia, Germany, and France

The three countries experienced two main pandemic waves. The first spread from January to July. The second one started around August and is still ongoing.

The first positive cases of COVID-19 were detected in France and Germany 3 days apart (24 January (France) and 24 January (Germany)), while the first case detected in Tunisia was on 2 March 2020 [14].

Since its introduction in the three countries, the pandemic has taken on different evolutionary aspects.

**Figure 1:**
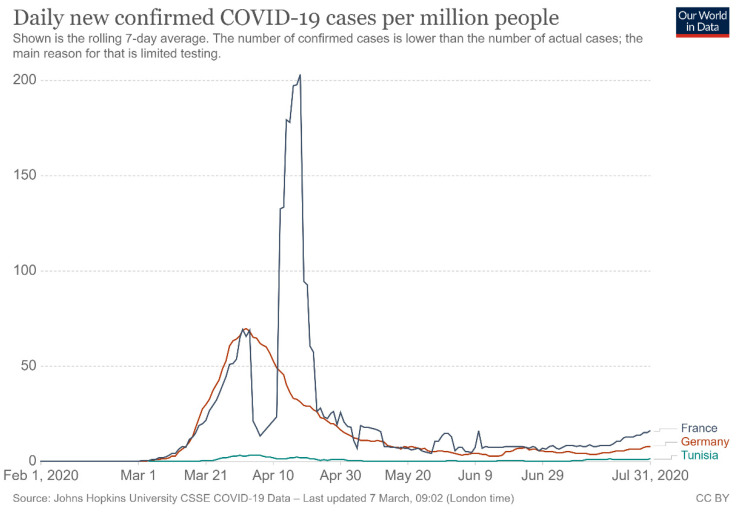
Shown is the rolling 7-day average of coronavirus cases in France, Germany, and Tunisia from 1 February 2020 to 31 July 2020. Source: Max Roser HR, Esteban Ortiz-Ospina, Joe Hasell. Posted online on OurWorldInData.org, 30 January 2021.

During the first pandemic wave, the curves relating to the evolution of the number of new positive cases (per million inhabitants per week) of France and Germany took paths in superimposable bells with peaks around 7 April. For Tunisia, the curve remained rather flat without exponential changes in the number of cases (Figure 1) [14].

**Figure 2:**
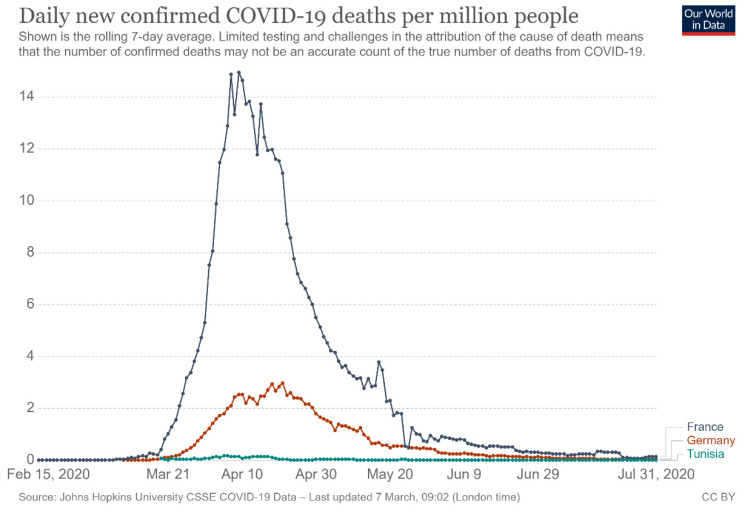
Shown is the rolling 7-day average of deaths caused by coronavirus in France, Germany, and Tunisia from 1 February 2020 to 31 July 2020. Source: Max Roser HR, Esteban Ortiz-Ospina, Joe Hasell. Posted online on OurWorldInData.org, 30 January 2021.

For France and Germany, the curves of the number of new deaths per million inhabitants (Figure 2) took the same shape as the curves of the new positive cases. They show a slight evolution initially followed by a rapid increase and then a relapse. The most striking observation is that the curves of France and Germany ceased to follow follow the same pattern. France recorded much higher death rates than Germany. This can be explained by the early and high level of testing in Germany among a wide sample of the population (which included milder cases in younger people). That means that more people with few or no symptoms were identified as COVID-positive, increasing the number of known cases, but not the number of fatalities [15].

Due to a delay in the transmission of death certificates and the consolidation of data, the French data for the week of 13 April until 19 April were specified the following week of 20–26 April, which explains the drop in data around 13 April [16].

For Tunisia, the number of deaths was so small that the curve is practically linear [14].

#### 1.1.3. The World Health Organization’s COVID-19 Strategic Plan and Monitoring and Evaluation Framework

On 14 April 2020, the WHO released an update of the *COVID-19 Preparedness and Response Strategic Plan* (PSPR) (released 3 February 2020) to inform the COVID-19 public health response at the national and sub-national levels and help identify gaps [4].

The eight pillars of the PSPR are:

1. National coordination, planning, and surveillance: Successful implementation of adaptive COVID-preparedness and response strategies will depend on the participation of the whole society in the plan and on the strength of national and sub-national coordination.
2. Risk communication and public engagement: Transparent communication to the public with responsive, empathetic, and culturally appropriate messages. Implementation of systems to detect and respond to concerns, rumors and false information.
3. Surveillance, early intervention, and case investigation: Early detection of imported cases, comprehensive and rapid contact tracing, and case investigation.
4. Ports of entry: Support for efforts and resources at ports of entry.
5. National laboratories: Preparation of laboratory capacity to manage the volume of COVID-19 testing.
6. Infection, prevention, and control: Review and improvement of infection prevention and control practices.
7. Case management: Preparation of health facilities and training of health professionals for the management of COVID-19 cases.
8. Logistics and operational support: Identification of resources and supply systems (supply, storage, security, transportation, and distribution).

On 5 June 2020, the WHO released *The Monitoring and Evaluation Framework of the Strategic Preparedness and Response Plan* (SPRP) [17] to track preparedness, responses, and situations during the COVID-19 pandemic. It was intended to assess performance and provide recorded information to support the analysis of progress against the *COVID-19 Preparedness and Response Strategic Plan* (PSPR) [4,17]. It includes input, output, and outcome indicators to achieve the objectives.

## 2. Materials and Methods

### 2.1. Study Type

This work consists of a study of the literature aimed at mapping systematically the different documentation related to the national strategies against COVID-19 in Tunisia, France, and Germany.

The procedure of systematic mapping warrants the organization in a systematized, clear, and robust manner, of references about a certain context, and assisting the decision-making process [18].

### 2.2. Eligibility Criteria

We note that, because this is a recent and rapid pandemic, scientific publications that discuss strategies are still scarce. For this reason, the study will also include official institutional documents published by the authorities of each country. 

The inclusion criteria were defined according to the PIS criteria [19]:

- P (population): the governments of the three countries concerned (France, Germany, Tunisia)
- I (intervention): COVID-19 national pandemic strategies and policies
- S (study design): Any document that meets the following criteria will be included:
  o  Any scientific article or official institutional document.
  o  Carried out before 31 July 2020.
  o  Languages: studies written in French, Arabic, German, or English.
  o  Concerns France, Germany, or Tunisia.

### 2.3. Information Sources

The following websites were used to search for scientific articles:

- The electronic database PubMed (https://pubmed.ncbi.nlm.nih.gov, accessed on 1 September 2020).
- Google Scholar for the search of grey literature.

The following websites were used to search for government institutional publications:

- The platform of the French Ministry of Solidarity and Health (https://solidarites-sante.gouv.fr/, accessed on 1 September 2020).
- The platform Santé Publique France”’ (https://www.santepubliquefrance.fr/, accessed on 1 September 2020).
- The French government website (https://www.gouvernement.fr/, accessed on 1 September 2020).
- The platform of the Robert Koch Institute (Germany) (https://www.rki.de/DE/Home/homepage_node.html, accessed on 1 September 2020).
- The platform of the Federal Ministry of Health Germany (https://www.bundesgesundheitsministerium.de/, accessed on 1 September 2020).
- The platform of the Ministry of Public Health Tunisia (http://www.santetunisie.rns.tn/, accessed on 1 September 2020).
- The National Observatory for New and Emerging Diseases (https://www.onmne.tn/fr/index.php, accessed on 1 September 2020).
- The website “COVID-19 Tunisia”, which was set up by the Presidency of the Tunisian government (https://covid-19.tn/fr/accueil-2, accessed on 2 September 2020).

### 2.4. Search Strategy

Keywords and search equations used to search for articles:

- 1#: “2019 nCoV” OR “2019nCoV” OR “2019 novel coronavirus” OR “COVID-19” OR COVID19 OR “new coronavirus” OR “novel coronavirus” OR “SARS CoV-2” OR “فيروس كورونا المستجد’’.
- 2#: “Tunisia” OR “France” OR “Germany” OR “Deutschland” OR “Tunisie” OR “Tunesien” OR “Frankreich” OR “Allemagne” OR “تونس” OR “ فرنسا” OR”ألمانيا “
- 3#: “Health Polic”[Mesh]” OR “Strategy” OR “ Strategie”; OR “ إستراتيجية “ OR “ Gesundheitspolitik”
- Equation de recherche (Pubmed): 1# AND 2# AND 3#
- Keywords used to search for institutional documents:
- For Tunisian websites: 2019 novel coronavirus/COVID-19/new coronavirus/novel coronavirus/SARS CoV-2/les coronavirus/la pandémie de covid-19/فيروس كورونا المستجد/كورونافيروس/وباء الكورونا
- For French websites: 2019 novel coronavirus/COVID-19/new coronavirus/novel coronavirus/SARS CoV-2/les coronavirus/la pandémie de covid-19/
- For German websites: 2019 novel coronavirus/COVID-19/new coronavirus/novel coronavirus/SARS CoV-2/Coronaviren/die Covid-19 Pandemie/

### 2.5. Study Records

The document selection process involved several essential steps based on the PRISMA 2009 model [20]:

- Identification by title: in a first step, the documents were identified according to their titles.
- Eligibility: the second identification was carried out on the summary of each bibliographic reference (this step concerned only scientific articles).
- Inclusion: based on the complete texts, by applying the pre-established criteria and after elimination of duplicates.

Between 1 September and 1 November 2020, two reviewers (KL, FH) independently screened the titles, abstracts, and, if ambiguous, full texts for the inclusion of documents. Discrepancies were resolved through discussions among the two reviewers, and in consultation with a third reviewer (RN), to reach a consensus. Subsequently, KL and FH independently conducted information extraction from the included documents. Discrepancies were similarly resolved through discussion among the reviewers and in consultation with NE, RN, and MRT.

### 2.6. Data Extraction

The results were extracted based on the *Monitoring and Evaluation Framework for the Strategic Plan for Preparedness and Response*, which was published on 5 June 2020 by the WHO [17]. This framework proposes global and national assessment indicators for each of the eight strategic pillars published by WHO in the *Strategic Plan for Preparedness and Response to COVID-19* (SPPR). Only national indicators were considered in extracting the results of our study.

### 2.7. Data Synthesis

An abductive approach was used in the analysis of the results. This approach is based on all of the pillars of the WHO’s *Strategic Preparedness and Response to COVID-19 Monitoring and Evaluation Framework* (SPRP) [21].

A narrative and mapping analysis of the results, which are presented in tabular and map form, is done.

## 3. Results

### 3.1. Quantitative Results

**Figure 3:**
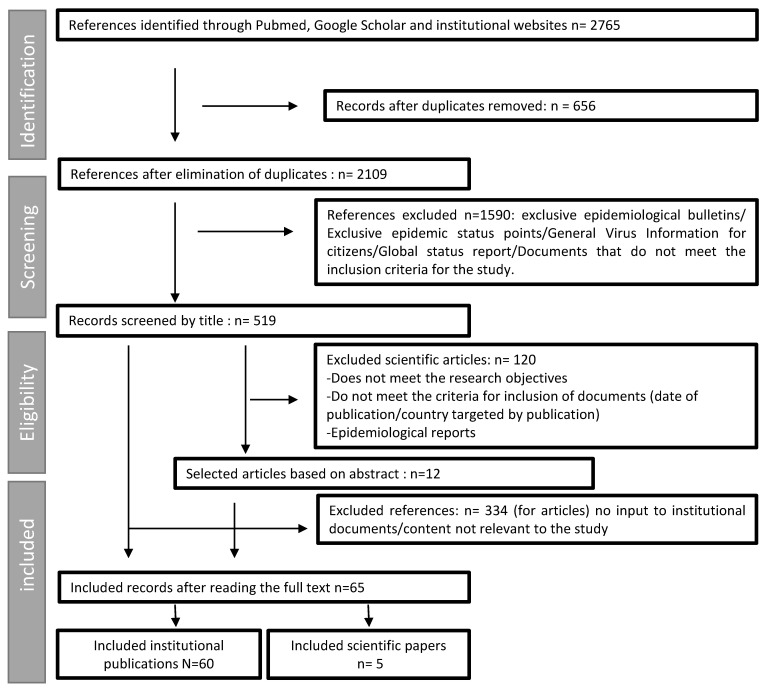
Flow diagram of the review process.

The search strategy identified 2765 potential documents, 1590 documents were eliminated based on the title, 120 articles were eliminated based on the abstract, and a total of 396 were retained for final selection. A number of 65 documents were included in the study, including 60 institutional and 5 scientific publications (Figure 3).

Among the documents included, only five are scientific articles, while 60 are institutional documents. Most institutional publications are devices/plans or guides (33.8%) and information sources for citizens (Table 2).

### 3.2. Qualitative Results

Table 3, Table 4, Table 5, Table 6, Table 7, Table 8, Table 9 and Table 10 illustrate the set of data selected based on the main strategic pillars of the WHO (which were studied using a set of indicators).

#### 3.2.1. Pillar 1: National Coordination, Planning, and Monitoring

The references selected for this first pillar show that the three countries have chosen similar measures and plans in national pandemic coordination, planning, and surveillance, including activation of a national response plan (based primarily on World Health Organization recommendations), and use of reviews and reporting to strengthen pandemic response, barrier measures, and general containment (Table 3). However, each plan was marked by different timelines and periods of implementation and set-up, which can be seen in Supplementary Material Table S1.

Germany distinguished itself from Tunisia and France by not declaring a state of emergency at the national level.

#### 3.2.2. Pillar 2: Risk Communication and Population Mobilization

The governments of the three countries have opted for transparent communication to the public. They have also put in place mechanisms to regularly gather community feedback and assess public perceptions and concerns, as well as practical and logistical support for people living in socially vulnerable contexts (Table 4).

No publications were found for Tunisia and France regarding the presence of a national communication plan on the risks related to COVID-19.

#### 3.2.3. Pillar 3: Surveillance, Rapid Response Teams, and Case Investigation

The three countries of the study have used surveillance systems to ensure regular epidemiological monitoring and to study the characteristics of the SARS-CoV2 virus.

Different testing strategies have been identified depending on the country and the period. Germany has distinguished itself from Tunisia and France by using mass screening since the beginning of the pandemic.

No publications were found regarding prevalence estimates of infection from prevalence studies in Tunisia (Table 5).

#### 3.2.4. Pillar 4: Points of Entry, International Travel, and Transport

International travel management strategies have been implemented in all three countries.

Tunisia has followed stricter measures in the confinement of passengers on repatriation flights during the period of border closure (Table 6).

#### 3.2.5. Pillar 5: National Laboratories

Some references showed different strategic measures for national laboratories to face the pandemic. No reference was found for Tunisia and France regarding the number of laboratories authorized until 31 July 2020.

The preparation of the laboratories in Tunisia has not been documented in the selected references (Table 7).

#### 3.2.6. Pillar 6: Infection Prevention and Control

Guidance documents for healthcare centers to implement a COVID-19 circuit have been identified in the three countries of the study.

Contact tracing strategies are different in each of the three countries (Table 8, Supplementary Material Table S9).

#### 3.2.7. Pillar 7: Case Management

The three studied countries have implemented case-management measures by training health professionals and by census of health facilities and preparing them for significant increases in the number of patients suspected of being infected with COVID-19 (Table 9).

#### 3.2.8. Pillar 8: Maintaining Essential Health Services and Systems

Continuity of care for non-COVID-19 patients was disrupted in the three countries (Table 10).

### 3.3. Synthesis of Results

**Figure 4:**
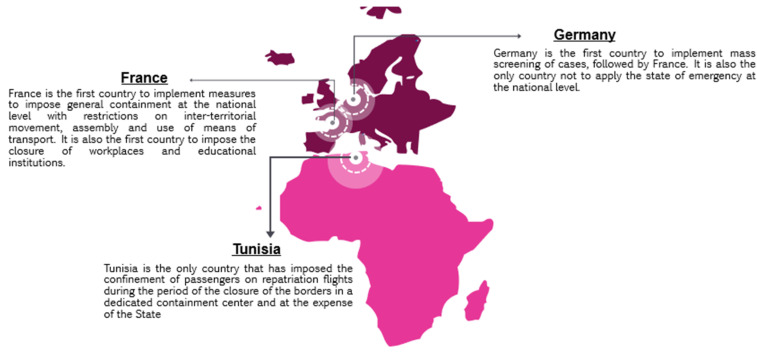
Mapping of the main differences in the national strategies of Tunisia, France, and Germany.

The measures taken in relation to the eight strategic pillars are for the most part similar for the three countries. However, there are differences in terms of the timing, the means put in place, and the methods used to implement certain measures (Figure 4).

## 4. Discussion

### 4.1. Discussion of the Method

The understanding of a national strategy is sometimes dependent on confidential data that is not accessible to all [73], which has led to a limited number of indicators for each pillar. However, the countries in the study chose transparency in communicating information to citizens and the world population.

It might have been interesting to compare the results through interviews with health leaders from each country, but as the crisis continued this was not possible.

### 4.2. Discussion of Results

#### 4.2.1. Impact of the Political Regime

The three countries have chosen similar measures and plans for national coordination, planning, and surveillance of the pandemic, including activation of a national response plan (based primarily on World Health Organization recommendations), use of reviews and reporting to strengthen the pandemic response, barrier measures, and general containment. However, each plan was marked by the nature of the political system in place.

While Tunisia and France have a centralized power and a common strategy for the entire country, Germany has been characterized by different strategies among the federal states, creating a more differentiated political set of actions across the country [89].

Indeed, the German government has been reluctant to make use of the provisions of the emergency constitutional powers [90]. The abuses of emergency powers in the last years of the Weimar Republic, which led to the rise and domination of Nazism, are still in their minds. These provisions included in the Basic Law of 1968 are still controversial and have never been used [91].

Nevertheless, on 27 March 2020, the Bundestag declared a national epidemic (“epidemische Lage von nationaler Tragweite”) on the legal basis of the Infection Protection Act, which gave national and subnational frameworks additional powers to combat the spread of COVID-19.

The federal level exercised its prerogatives regarding travel restrictions and closing borders, ensuring the availability of relevant health resources through the establishment of means for rapid acceleration of production. The states took measures at the local level, such as policies related to schools, kindergartens, and universities, as well as those related to business. This decentralization of power allowed the measures taken to be better adapted to local pandemic conditions and the specific needs of each federal state [92].

In addition to the duty to respond, a kind of constructive rivalry has developed between the Ländern and intensified during the fourth phase of crisis management, with several governments surpassing themselves and trying to do their best in developing strategies to revive public life [90].

On the French side, this decentralization has not found a place. The French authorities have chosen a common management policy based on an analysis of the evidence. Therefore, they created an advisory board of 11 scientists to help them manage the crisis. This approach carries with it a radical uncertainty, because the simple fact of examining the evolution of confirmed cases with a disease that spreads faster than influenza and has a higher morbidity rate, does not allow lessons to be learned in real time and decisions and policies to be made quickly [93].

Strict measures were taken by the three countries to control and limit gatherings, but these were sometimes contradicted by other decisions. In France, for example, despite the ban on rallies, the first round of national elections on 15 March was maintained, thus confronting the population with a situation of double constraint by dissonant incentives [94].

#### 4.2.2. Testing/Screening Strategy and Tracing

Germany’s testing strategy is distinguished from those in France and Tunisia by the early launch of mass population screening campaigns. This strategy, which meets the recommendations of the World Health Organization, has been practiced by several other countries such as South Korea and Australia [10].

France chose the strategy of targeted testing at the beginning of the pandemic due to logistical problems, the limited number of accredited laboratories (only 45 in public institutions), and the limited availability of SARS-COV-2 reagents for RT-PCR at the beginning of the pandemic [94]. However, France rapidly increased its testing capacity from 47,732 tests a day on 30 March 2020 to 968,454 tests a day on 30 May 2020 [37].

Tunisia chose the same strategy of targeted testing as in France: tests were carried out mainly for people who were in contact with people infected with COVID-19 or patients with symptoms. However, the capacity for laboratory analysis (RT PCR) in Tunisia is very limited compared to France [95].

#### 4.2.3. Importance of Prevention and Predictive Measures

With the delayed appearance of the pandemic in Tunisia, the newly elected Tunisian government chose to learn from countries that have already reached the exponential phase of the spread of the infection, as well as from those that have lived through and gone beyond this phase. The Tunisian people who saw the consequences and effects of the pandemic in Italy, Spain, and China quickly joined the government effort and showed absolute solidarity [95].

Indeed, the lack of resources in Tunisia was remedied by its predictive strategy, which made it possible to take strict and effective measures, such as measures to contain passengers on repatriation flights during the period of the closure of the borders.

The Tunisian containment strategy has distinguished itself from its two European counterparts by the mandatory confinement at the expense of the state in a dedicated center. This strategy has proven to be effective because at this phase of the pandemic, the risk of COVID-19 occurring among arrivals after the general quarantine was 60 times greater than the incidence rate at the premises. That means that the risk of local infection was mainly related to imported cases, according to the Tunisian Ministry of Health [96].

The effectiveness of Tunisia’s predictive strategy at the beginning of the pandemic can be seen through the relapse and flattening of its epidemiological curve relative to the number of new cases from the end of April until the month of July [36].

Indeed, the application of containment measures in the world varies clearly between countries. Few countries have chosen zero containment, with the exception of South Korea, Taiwan, and the Netherlands, in order to establish collective immunity. Other countries, such as the United Kingdom, initially opted for this strategy but changed their strategy in view of the rapid spread of the virus and the growing social challenges [95].

France and Germany followed a model of partial containment at the beginning of the crisis, by the isolation of epidemic outbreaks at regional level, and by the closure of schools/universities and non-essential public places.

#### 4.2.4. Social Acceptance and Commitment

The three countries made use of new information and communication techniques, through the adoption of mobile applications for case-finding and tracing. However, their effectiveness has been challenged because of the delicate balance between protecting public health and respecting fundamental rights such as privacy [97,98].

Thus, despite the urgency of the context, certain measures taken by the authorities have been challenged, and judged as an attack on democracy and especially on individual freedoms. Demonstrations and protest movements were organized to denounce decisions taken, some even tried and won trials before the courts [90].

Moreover, the social acceptance of these measures varied according to the culture, the political regime in place and the epidemic history of the country. Greater acceptance has been found in areas of the world that have faced previous epidemics (including SARS) [95].

Noting that, for Tunisia, social engagement has played a key role in its fight against the pandemic. The Tunisian government has chosen the transparency route since the beginning (regular speeches by the Ministry of Health, a permanent government presence on TV sets, a daily press briefing, etc.). This has stimulated social engagement and mobilized civil society to support the government’s efforts. A collection of donations took place through a fund, called 1818, in which financial contributions were deposited [89].

#### 4.2.5. Continuity of Non-COVID-19 Care Services

There was a disruption in continuity of care for non-COVID-19 patients in all three countries. This disturbance can be explained by two phenomena. On the one hand, hospitals deprogrammed their activities and postponed their consultations/operations massively in order to have the resources and equipment for the management of the epidemic crisis. On the other hand, hospital arrivals for non-COVID-19 conditions declined for a variety of reasons, such as not wanting to “disturb” the staff while they were overwhelmed by COVID-19 case management, fear of travelling to the hospital, or rescheduling appointments that were not considered urgent (screening, vaccination, etc.) [25].

## 5. Conclusions

The COVID-19 pandemic is neither the first nor the last viral pandemic that societies around the world have been, are, and will be affected by.

The first measures taken against the pandemic are of particular importance because they make it possible to slow down the spread and thus allow countries to gain a margin of time to prepare their material and human resources for defense.

Measures that have been shown to be effective in response strategies should be considered as future recommendations at an international level (such as early detection, mass screening, effective management logistics, protection of the vulnerable population, etc.). Indeed, the COVID-19 pandemic has been a difficult ordeal for humanity, which is called upon to review its priorities, through the highlighting of the health and hygiene dimensions. These dimensions need much more attention and investment.

National strategy studies, which include countries with different health systems (e.g., the Beveridge model), as well as those that include the pandemic in its four waves, are needed to gain a more precise overview of control strategies.

## Figures and Tables

**Table 1:** Demographic data relevant to the impact of COVID-19 in Tunisia, Germany, France.

	Tunisia	Germany	France
Population in millions 2019	11.57	82.91	58.24
Government type	Parliamentary republic	Federal parliamentary republic	Republic, semi-presidential
Life expectancy at birth in years (2018)	77	81	83
% Pop. over 65 years old (2019)	9%	22%	20%
Prevalence of smoking (15 years old and over)	32.7 (2016)	30.6 (2016)	32.7 (2016)

**Table 2:** Types of documents included in the study.

Type of Documents	References	Number	Percentage	
Scientific articles	[22,23,24,25,26]	5	7.7%	
**Institutional Records**				
Decrees	[27,28,29,30]	4	6.6%	92.3%
Web pages	[31,32,33,34,35,36,37,38]	8	12.3%	
Devices, guides, and plans	[39,40,41,42,43,44,45,46,47,48,49,50,51,52,53,54,55,56,57,58,59,60]	22	33.8%	
Articles	[61,62]	2	3.1%	
Interview reports	[63,64,65]	3	4.6%	
Press releases	[66,67]	2	3.1%	
Situation points	[68,69,70]	3	4.6%	
Information for citizens	[71,72,73,74,75,76,77,78,79,80,81,82,83,84,85,86]	16	24.6%	
Total		65	100%	

**Table 3:** Pillar 1: National coordination, planning, and monitoring.

Indicator	Tunisia	France	Germany
1.1 Availability of a trigger for the activation and deactivation of a pandemic emergency response (and mechanism for updating)	Yes [28]	Yes [41]	Yes [30]
1.2 Activation of a national response plan	Yes [40,71]	Yes [26,71]	Yes [38,40]
1.3 National state of emergency	Yes [40,71]	Yes [21]	No [21]
1.4 Use of reviews to strengthen the pandemic response	Yes [28,71]	Yes [43,74]	Yes [31]
1.5 General lockdown (variation over time (Supplementary Material Table S1))	Yes [23]	Yes [23]	Yes [23]
1.6 Recommended physical distance between individuals in public spaces	Yes [75]	Yes [44]	Yes [63]
1.7 Closure of public spaces (non-essential shops, restaurants, etc.)	Yes [76]	Yes [72]	Yes [22,24]
1.8 Restrictions of the use of public transport (variation over time (Supplementary Material Table S2))	Yes [23]	Yes [23]	Yes [23]
1.9 Closure of workplaces (variation over time (Supplementary Material Table S3))	Yes [23]	Yes [23]	Yes [23]
1.10 Teleworking	Yes [77]	Yes [29]	Yes [45]
1.11 Closure of educational institutions (variation over time (Supplementary Material Table S4))	Yes [23]	Yes [23]	Yes [23]
1.12 Interventions in place for risk groups and vulnerable population	Yes [78]	Yes [46]	Yes [47]
1.13 Obligation to use face masks in the community in closed spaces	Yes [75]	Yes [72]	Yes [79]
1.14 National movement restrictions (variation over time (Supplementary Material Table S5))	Yes [23]	Yes [23]	Yes [23]
1.15 Public gatherings restrictions (variation over time (Supplementary Material Table S6))	Yes [23]	Yes [23]	Yes [23]

**Table 4:** Pillar 2: Risk communication and community engagement.

Indicator	Tunisia	France	Germany
2.1 COVID-19 risk communication and community engagement (RCCE) plan in place	No data found	No data found	Yes [39,41]
2.2 Mechanisms in place to routinely capture community feedback and assess public perceptions, concerns, and trust	Yes [48,71]	Yes [32,49]	Yes [68]
2.3 Mechanisms in place to provide practical and logistical support to people living in socially vulnerable settings	Yes [80]	Yes [46,49]	Yes [39,66]

**Table 5:** Pillar 3: Surveillance, rapid response teams, and case investigation.

Indicator	Tunisia	France	Germany
3.1 Surveillance systems in place for comprehensive monitoring of COVID-19 epidemiology	Yes [28]	Yes [42]	Yes [31]
3.2 Monitoring of SARS-CoV-2 virus characteristics	Yes [28]	Yes [42]	Yes [31]
3.3 Estimates of infection prevalence from prevalence studies	No data found	Yes [61]	Yes [69]
3.4 Testing strategies	Variation over time (Supplementary Material Table S7) [23]		
3.5 Monitoring of new confirmed cases by sex and age groups	Yes [36,40]	Yes [37,73]	Yes [31,38]
3.6 Monitoring the number of probable and confirmed deaths due to COVID-19 and classification by sex and age groups	Yes [36,40]	Yes [37,73]	Yes [31,38]
3.7 Availability of mobile app(s) to complement manual contact tracing	E7mi [33]	Stopcovid [34]	Corona warn app [35]

**Table 6:** Pillar 4: Points of entry (PoE), international travel, and transport.

Indicator	Tunisia	France	Germany
4.1 Presence of a public health emergency plan in PoE	Yes [30,70]	No data found	Yes [41]
4.2 International travel management	Variation over time (Supplementary Material Table S8)		
	[23,60,70]	[23]	[23]
4.3 Containment strategy of passengers on repatriation flights during the border closure period.	Total closure of borders: 16 March [81] — Quarantine, in dedicated centers, enforced by the government, for all arrival passengers from abroad: 21 March–05 June [67,86] — Quarantine, in dedicated centers at the expense of arrival passengers from abroad: 5 June–27 June [82] — Border reopening 27 June [83]	Auto-quarantine recommended [64]	No data found

**Table 7:** Pillar 5: National laboratories.

Indicator	Tunisia	France	Germany
5.1 Preparation of laboratory capacity to manage large-scale COVID-19 testing (within the country or through international agreements)	No data found	Yes [50,72]	Yes [51]
5.2 Laboratory numbers allowed until July 31	No data found	No data found	69 [87]
5.3 Communication of number of people tested for COVID-19 per week	Yes [36,40]	Yes [37,73]	Yes [31,38]

**Table 8:** Pillar 6: Infection prevention and control.

Indicator	Tunisia	France	Germany
6.1 Recommendation to have a clinical referral system in place to care for COVID-19 cases	Yes [52]	Yes [50,62]	Yes [53]
6.2 Contact tracing strategy (variation over time (Supplementary Material Table S9))	YES [24]	Yes [23]	Yes [23]

**Table 9:** Pillar 7: Case management.

Indicator	Tunisia	France	Germany
7.1 Clinical guidance guide for treating COVID-19 patients	Yes [54]	Yes [50,55,56,88]	Yes [57]
7.2 Isolation of confirmed and probable COVID-19 cases	Yes [40]	Yes [58,59]	Yes [39]
7.3 Training healthcare professionals to manage COVID-19 patients	Yes [84]	Yes [27,50]	Yes [39]

**Table 10:** Pillar 8: Maintaining essential health services and systems.

Indicator	Tunisia	France	Germany
8.1 Maintaining of the vaccination coverage during the crisis	Maintained [85]	No data found	No data found
8.2 Continuity of care for non-COVID-19 patients	Partial [85]	Partial [25]	Partial [26,65]

## Data Availability

Not applicable.

## Supplementary Materials

The following are available online at https://www.mdpi.com/article/10.3390/ijerph18168622/s1, Table S1: Indicator 1.5 General Lockdown, Table S2: Indicator 1.8 Restrictions on the use of public transport, Table S3: Indicator 1.9 Closure of workplaces, Table S4: Indicator 1.11 Closure of educational establishments, Table S5: Indicator 1.14 Restrictions on inter-territorial movements, Table S6: Indicator 1.15 Restrictions on gatherings, Table S7: Indicator 3.4 Testing strategies, Table S8: Indicator 4.2 Management of international travel, Table S9: Indicator 6.2 Contact tracing strategy.
