# Supplementary material for: The Early Stage of the COVID-19 Outbreak in Tunisia, France, and Germany: A Systematic Mapping Review of the Different National Strategies
Source: Int J Environ Res Public Health. 2021 Aug 15;18(16):8622. doi: 10.3390/ijerph18168622 (PMC8391965; doi:10.3390/ijerph18168622)
Supplement: Supplementary file 1 [file ijerph-18-08622-s001.zip › Supplementary materials.pdf]

Supplementary materials:

Table S1. Indicator 1.5 General Lockdown

Tab : Indicator 1.5 General Lockdown

**Country**	01/02	09/03	17/03	20/03	21/03	06/05	11/05	15/05	08/06	22/06	31/07
**Germany**	0	1	1	1	2	0	0	0	0	0	0
**France**	0	0	2	2	2	2	1	1	1	0	0
**Tunisia**	0	0	0	2	2	2	2	1	0	0	0

0 - no mesure

1 - Recommend not to leave the house

2 - Require not to leave the house with exceptions for daily exercise, shopping and “essential” travel

Table S2. Indicator 1.8 Restrictions on the use of public transport

Tab : Indicator 1.8 Restrictions on the use of public transport						
**Country**	01/02	16/03	22/03	08/06	22/06	31/07
**Germany**	0	0	0	0	0	0
**France**	0	1	1	1	0	0
**Tunisia**	0	0	1	0	0	0

0 - No measurement

1 - Recommend closure (or considerably reduce the volume / route / means of transport available)

2 - Require closure (or prohibit most citizens from using it)

Table S3. Indicator 1.9 Closure of workplaces

Tab : Indicator 1.9 Closure of workplaces									
**Country**	01/02	17/03	18/03	22/03	04/05	11/05	08/06	22/06	31/07
**Germany**	0	0	0	2	2	2	2	2	2
**France**	0	3	3	3	3	2	2	1	1
**Tunisia**	0	0	1	3	2	2	0	0	0

0 - No measurement

1 - Recommend closure (or work from home)

2 - Require closure (or work from home) for certain sectors or categories of workers

3 - Require the closure (or working from home) of all workplaces except essential workplaces (for example: grocery stores, doctors)

Table S4. Indicator 1.11 Closure of educational establishments

Tab : Indicator 1.11 Closure of educational establishments													
**Country**	01/02	26/02	02/03	09/03	13/03	16/03	04/05	11/05	28/05	18/06	22/06	07/07	31/07
**Germany**	0	2	2	2	2	3	2	2	2	3	3	1	1
**France**	0	0	3	3	3	3	3	2	2	2	0	0	0
**Tunisia**	0	0	0	2	3	3	3	3	2	2	2	2	2

0 - No measurement

1 - Recommend closure

2 - Require closure (only certain levels or categories, for example just high school, or just public schools)

3 - Require the closure of all levels

Table S5. Indicator 1.14 Restrictions on inter-territorial movements

**Tab : indicator 1.14 Restrictions on inter-territorial movements**									
**Country**	01/02	14/03	17/03	18/03	19/03	20/03	04/06	22/06	31/07
**Germany**	0	0	0	1	2	2	2	2	2
**France**	0	1	2	2	2	2	2	1	1
**Tunisia**	0	0	0	0	0	2	0	0	0

0 - No measurement

1 - Recommend the restriction of movement

2 - Restrict movement

Table S6. Indicator 1.15 Restrictions on gatherings

Tab : Indicator 1.15 Restrictions on gatherings

Country	01/02	29/02	10/03	20/03	21/03	11/05	08/06	07/07	31/07
**Germany**	0	0	1	1	4	4	4	3	4
**France**	0	4	4	4	4	3	3	3	3
**Tunisia**	0	0	0	4	4	4	0	0	0

0 - No restriction

1 - Restrictions on very large gatherings (over 1000 people)

2 - Restrictions on gatherings between 100-1000 people

3 - Restrictions on gatherings of 10 to 100 people

4 - Restrictions on gatherings of less than 10 people

Table S7. Indicator 3.4 Testing strategies

Tab : Indicator 3.4 Testing strategies								
**Country**	01/02	25/02	02/03	17/03	29/04	11/05	25/07	31/07
**Germany**	1	1	1	1	3	3	3	3
**France**	0	2	2	1	1	2	3	3
**Tunisia**	0	0	1	1	1	1	1	1

0 - No testing policy

1 - Only those who both (a) have symptoms AND (b) meet specific criteria (for example, key workers admitted to hospital have been in contact with a known case, have returned from there 'foreign)

2 - Anyone with symptoms of COVID 19

3 - Open public testing (e.g. drive-thru testing available for asymptomatic people)

Table S8. Indicator 4.2 Management of international travel

Tab : Indicator 4.2 Management of international travel										
**Country**	01/02	28/02	04/03	09/03	16/03	17/03	18/03	16/05	27/06	31/07
**Germany**	0	1	1	1	3	3	4	3	3	3
**France**	2	2	2	2	2	3	3	3	3	3
**Tunisia**	0	0	1	2	4	4	4	4	3	3

0 - No measurement

1 - Screening

2 - Arrivals in quarantine from high-risk regions

3 - Prohibition of high-risk regions

4 - Total closure of borders

Table S9. Indicator 6.2 Contact tracing strategy

Tab : Indicator 6.2 Contact tracing strategy								
**Country**	01/02	26/02	02/03	18/03	07/05	11/05	12/06	15/06
**Germany**	2	2	2	1	1	1	1	2
**France**	0	1	1	1	1	2	2	2
**Tunisia**	0	0	1	1	2	2	2	2

0 - No contact tracking

1 - Limited contact search - not performed in all cases

2 - Complete contact search - performed for all cases
